# Predicting porosity, permeability, and tortuosity of porous media from images by deep learning

**DOI:** 10.1038/s41598-020-78415-x

**Published:** 2020-12-08

**Authors:** Krzysztof M. Graczyk, Maciej Matyka

**Affiliations:** Institute of Theoretical Physics, Faculty of Physics and Astronomy, University of Wrocław, pl. M. Borna 9, 50-204 Wrocław, Poland

**Keywords:** Fluid dynamics, Hydrology

## Abstract

Convolutional neural networks (CNN) are utilized to encode the relation between initial configurations of obstacles and three fundamental quantities in porous media: porosity ($\varphi$), permeability (*k*), and tortuosity (*T*). The two-dimensional systems with obstacles are considered. The fluid flow through a porous medium is simulated with the lattice Boltzmann method. The analysis has been performed for the systems with $\varphi \in (0.37,0.99)$ which covers five orders of magnitude a span for permeability $k \in (0.78, 2.1\times 10^5)$ and tortuosity $T \in (1.03,2.74)$. It is shown that the CNNs can be used to predict the porosity, permeability, and tortuosity with good accuracy. With the usage of the CNN models, the relation between *T* and $\varphi$ has been obtained and compared with the empirical estimate.

## Introduction

Transport in porous media is ubiquitous: from the neuro-active molecules moving in the brain extracellular space^1,2^, water percolating through granular soils^3^ to the mass transport in the porous electrodes of the Lithium-ion batteries^4^ used in hand-held electronics. The research in porous media concentrates on understanding the connections between two opposite scales: micro-world, which consists of voids and solids, and the macro-scale of porous objects. The macroscopic transport properties of these objects are of the key interest for various industries, including healthcare^5^ and mining^6^.

Macroscopic properties of the porous medium rely on the microscopic structure of interconnected pore space. The shape and complexity of pores depend on the type of medium. Indeed, the pores can be elongated and interwoven showing a high porosity and anisotropy like in fibrous media^7,8^. On the other hand, the medium can be of low porosity with a tight network of twisted channels. For instance, in rocks and shales, as a result of erosion or cracks, large fissures can be intertwined in various ways^6^.

Porosity ($\varphi$), permeability (*k*), and tortuosity (*T*) are three parameters that play an important role in the description and understanding of the transport through the porous medium. The porosity is the fundamental number that describes the fraction of voids in the medium. The permeability defines the ability of the medium to transport fluid. Eventually, the tortuosity characterizes the paths of particles transported through the medium^9,10^. The porosity, permeability, and tortuosity are related by the Carman-Kozeny^11^ law, namely,

1 $$\begin{aligned} k=\frac{\varphi ^3}{cT^2S^2}, \end{aligned}$$

where *S* is the specific surface area of pores and *c* is the shape factor. The tortuosity is defined as the elongation of the flow paths in the pore space^12,13^:

2 $$\begin{aligned} T=\frac{L_\text {eff}}{L}, \end{aligned}$$

where $L_\text {eff}$ is an effective path length of particles in pore space and *L* is a sample length ($L<L_{eff}$ and thus, $T>1$). The *T* is calculated from the velocity field obtained either experimentally or numerically. There are experimental techniques for particle path imaging in a porous medium, like particle image velocimetry^14^. However, they have limitations due to the types of the porous medium. There are two popular numerical approaches for estimation of *T*. In the first, the particle paths are generated by the integration of the equation of motion of fluid particles^11,12^. In the other, used in this work, *T* is calculated directly from the velocity field by averaging its components^11,15^. Indeed, the tortuosity is given by the expression

3 $$\begin{aligned} T=\frac{\langle u\rangle }{\langle u_x\rangle }, \end{aligned}$$

where $\langle u\rangle$ is the average of the magnitude of fluid velocity and $\langle u_x\rangle$ is the average of its component along macroscopic direction of the flow^15^. The pedagogical review of the approach is given by Matyka and Koza^16^, where the main features of the approach are explained in Figs. 3 and 5.

Calculating the flow in the real porous medium, with the complicated structure of pores, requires either a special procedure for grid generation in the standard Navier-Stokes solvers^17^ or usage of a kind of mesoscopic lattice gas-based methods^18^. Nevertheless of the type of solver used for the computation, the simulation procedure is time and computer resource consuming. Thus we propose the convolutional neural networks (CNN) based approach to simplify and speed up the process of computing the basic properties of a porous medium.

The deep learning (DL)^19^ is a part of the machine learning and artificial intelligence methods. It allows one to analyze or describe complex data. It is useful in optimizing the complicated numerical models. Eventually, it is a common practice to use the DL in problems not described analytically. The DL finds successful applications to real-life problems^20^ like automatic speech recognition, visual-image recognition, natural language processing, medical image analysis, and others. The DL has become an important tool in science as well.

Recently, the number of applications of the DL methods to problems in physics and material science grows exponentially^21^. One of the standard applications is to use the machine learning models to analyze the experimental data^22^. The deep neural networks are utilized to study the phase structure of the quantum and classical matters^23^. The DL is applied to solve the ordinary differential equations^24–26^. There are applications of the DL approaches to the problems of fluid dynamics. Here, the main idea is to obtain the relations between the system represented by the picture and the physical quantities, like velocities, pressure, etc.

The DL is one of the tools considered in the investigation of the transport in a porous medium. For instance, the neural networks are used to obtain porous material parameters from the wave propagation^27^. The permeability is evaluated using the multivariate structural regression^28^. It is obtained directly from the images^29^ too. The fluid flow field predictions by CNNs, based on the sphere packing input data, are given by Santos et al.^30^. The advantages of the usage of the machine learning techniques over physical models, in computing permeability of cemented sandstones, are shown by Male et al.^31^. The predictions of the tortuosity, for unsaturated flows in porous media, based on the scanning electron microscopy images, have been given by Zhang et al.^32^. Eventually, the diffusive transport properties have been investigated with CNNs, which take for the input the images of the media geometry only^33^.

Our goal is to find the dependence between the configurations of obstacles, represented by the picture, and the porosity, permeability, and tortuosity. The relation is going to be encoded by the convolutional neural network, which for the input takes the binary picture with obstacles and it gives, as an output, the vector $(\varphi , k, T)$. To automate, simplify, and optimize the process we use the state of the art of CNNs and DL techniques combined with the geometry input and fluid solver based outputs. Namely, to train the networks, we consider a large number of synthetic, random, porous samples with controllable porosity. From various models and types of complex phenomena related to porous media flow like multi-phase^18^, multi-scale^34^, regular^35^, irregular^7^ or even granular^36^ media we chose the model of single-phase fluid flow through randomly deposited overlapping quads—the single-scale porous media model with controllable porosity. The training data set contains the porous media geometries with corresponding numerical values of the porosity, permeability, and tortuosity. The two latter variables are obtained from the flow simulations done with the lattice Boltzmann solver.

As a result of the analysis, we obtain two CNN models which predict the porosity, permeability, and tortuosity with good accuracy. The relative difference between the predictions and the ’true’ values, for our best model, does not exceed $6 \%$. Eventually, we generate a blind set of geometry data for which we make predictions of the tortuosity and porosity. The obtained $T(\varphi )$ dependence is in qualitative agreement with the empirical fits.

The paper is organized as follows: in “Lattice Boltzmann method” section the lattice Boltzmann method is introduced, “Deep learning approach” section describes the CNN approach and the method of the data generation, whereas “Results and summary” section contains the discussion of the numerical results and summary.

## Lattice Boltzmann method

The data set consists of the rectangular pictures of the configuration of obstacles with corresponding quantities (labels): $\varphi$, *k*, and *T*, which are calculated from the lattice Boltzmann method (LBM) flow simulations.

The LBM has confirmed its capability to solve complex flows in complicated porous geometries^37^. It is based on the density distribution function, which is transported according to the discrete Boltzmann equation. In the single relaxation time approximation the transport equation reads:

4 $$\begin{aligned} \frac{\partial f}{\partial t} + \vec {v}\cdot \nabla f = - \frac{1}{\tau }(f-g), \end{aligned}$$

where $f(\vec {r},t)$ is the density distribution function, $\vec {v}$ is the macroscopic velocity, $\tau$ is the relaxation time and *g* is the Maxwell-Boltzmann distribution function at given velocity and density (see Duda et al.^15^ and He and Lu^38^).

**Figure 1:**
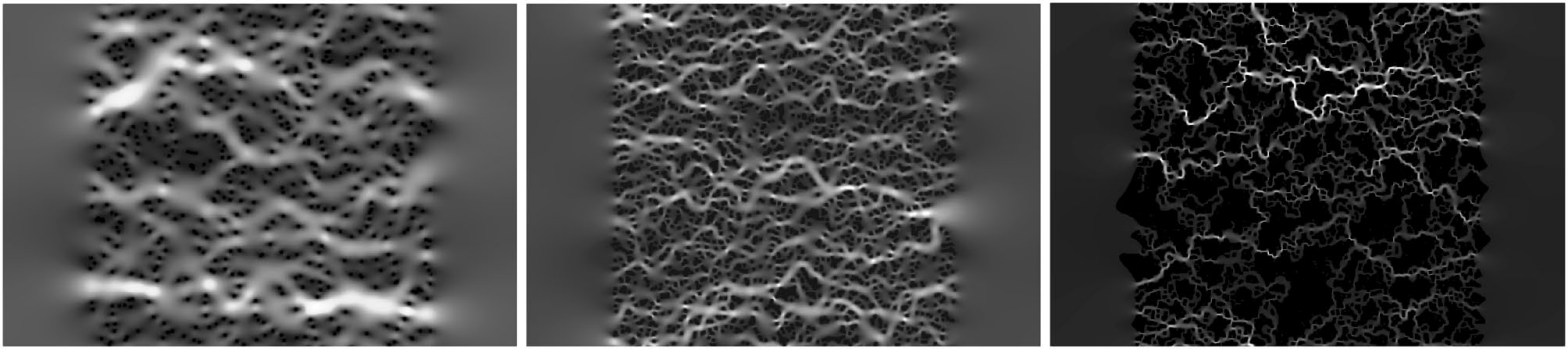
The exemplary pore-scale velocity magnitude in the fluid flow calculated using the LBM based on configurations from Fig. 2—brighter the color larger velocity. Contrast and brightness are adjusted to visualize the structure of emerged flow paths.

The LBM solver provides information about the time evolution of the density function, which is used to evaluate the velocity field. We consider the pore-scale approach—the obstacles are completely solid, while the pores are completely permeable. Hence the velocity field is solved at the pore space. Each simulation starts with the zero velocity condition. The sample is exposed to an external gravity force that pushes the flow. Notice that we keep the number of iterations larger than 10,000 and less than 1,000,000. For the steady-state condition, we take the sum of the relative change of the velocity field in the pore space:

5 $$\begin{aligned} c = \sum _{i,j} \frac{(u_{i,j}-u_{i,j}')^2}{u_{i,j}^2}, \end{aligned}$$

where *u* is the local velocity at the current time step (taken at nodes *i*,*j*), and $u'$ is the velocity at time step 500 times steps earlier. The local change of the velocity is monitored to verify if the steady-state is achieved. The exemplary velocity field resulting from the above procedure is shown in Fig. 1.

## Deep learning approach

### Convolutional neural networks

The convolutional neural networks are used to code the information about the dependence between the initial configuration of obstacles, represented by the picture, and the porosity, tortuosity as well as permeability. The CNN is a type of deep neural network designed to analyze multi-channel pictures. It has been successfully applied for the classification^39^ and the non-linear regression problems^40^.

The CNN consists of a sequence of convolutional blocks and fully connected layers. In the simplest scenario, a single block contains a convolutional layer with several kernels. Usually, the pooling layer follows on the convolutional layer, while in the hidden layer, the rectified linear units (ReLU)^40^ are considered, as the activation functions. The theoretical foundations of the convolutional networks are given by Goodfellow et al.^41^.

A kernel extracts a single feature of the input. The first layer of the CNN collects the simplest objects (features) such as edges, corners, etc. The next layers relate extracted features. The role of the pooling layer is to amplify the signal from the features as well as to reduce the size of the input. Usually, a sequence of fully connected layers follows on the section of the convolutional blocks.

The CNNs considered in this work contain the batch normalization layers^42^. This type of layer has been proposed to maintain the proper normalization of the inputs. It has been shown that having the batch normalization layers improves the network performance on the validation set. Moreover, it is a method of regularization, which is an alternative to the dropout technique^43^.

### Data

We consider the random deposition model of a porous medium. It is the popular method for generating porous structures for numerical solvers^44,45^. The samples are build of overlapping quad solids laying on the two-dimensional surface.

**Figure 2:**
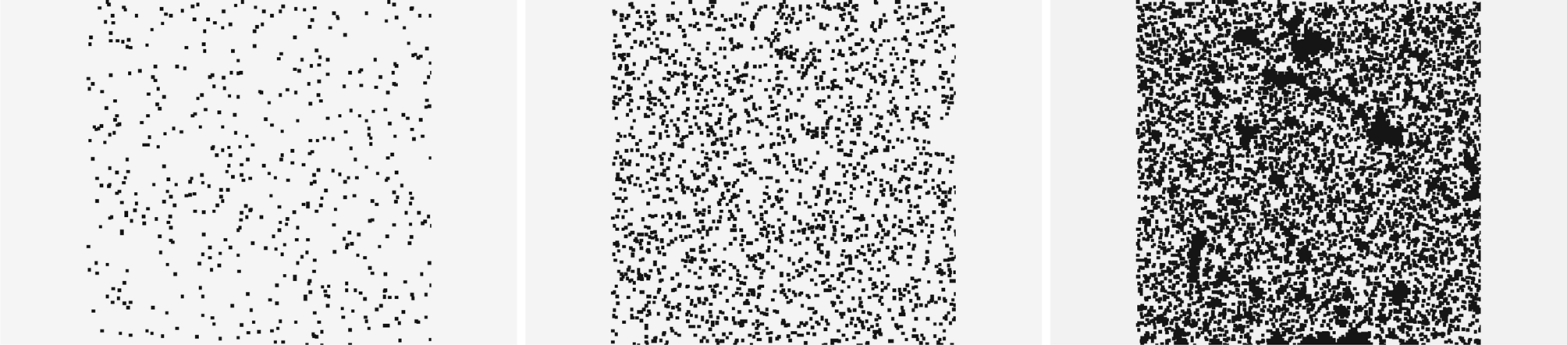
Exemplary random porous samples at porosity $\varphi =0.95, 0.8$ and 0.5 (from left to right), size $800\times 400$. Black blocks represent obstacles for the flow, while the interconnected light gray area is the pore space filled by fluid. Black clusters, visible for $\varphi =0.5$, are the effect of the filling-gap algorithm used for prepossessing of the data, after generating with random deposition procedure. The gaps are not accessible for the fluid and, thus, we fill them before they are provided to the fluid solver and neural network.

A total number of 100,000 one-channel figures with obstacles and predicted values of the porosity, tortuosity, and permeability have been prepared. A given figure, see Fig. 2, is the binary picture of the size $800\times 400$. It includes two vertical margins of the width 200 (pixels) each. The margins are kept to reduce the influence of boundaries on the calculations of the porosity, tortuosity, and permeability. But, for the CNN analysis, they do not contain the information. Hence in the training and inference process, the picture without margins is taken as an input for the network. Effectively, the input has the size $400\times 400$.

Each figure consists of $400 \times 400$ nodes, which are either free or blocked for the fluid flow. The periodic boundary condition is set at sides with margins, whereas two remaining sides are considered no-slip boundary condition. To generate the medium at given porosity, we start with an empty system and systematically add $4\times 4$ quad obstacles at random positions in the porous region. The margins are excluded from this deposition procedure. We do not consider blocking of sites, thus, obstacles may overlap freely. Each time an obstacle is placed, we update current porosity and stop, if the desired value is reached. In the next step of the generation of figures, we use the simple flood-filling algorithm to eliminate all the non-physical islands from each sample—Island is the pore volume completely immersed in solids. The exemplary porous samples, at three different porosity values, are shown in Fig. 2. Having the binary images of the pore space, the LBM solver calculates the velocity distribution from which the quantities of interest: the porosity, tortuosity, and permeability are obtained.

**Figure 3:**
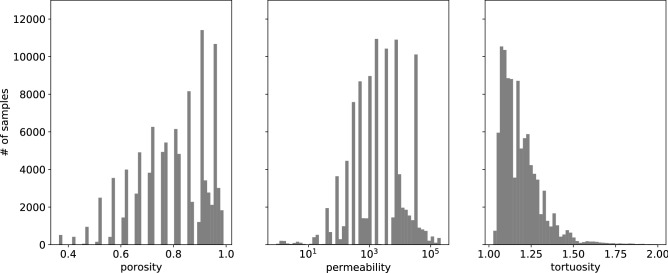
The distribution of the generated samples (training and validation data sets together) with respect to porosity, permeability, and tortuosity.

The success of the training of the network is determined by the quality of the data, its representative ability. The data should uniformly cover the space of label parameters. The ranges of the porosity, permeability, and tortuosity, for the systems discussed in our analysis, are given in Table 1. In the initial stage of the system generation procedure, the porosity of samples has been chosen from the uniform distribution. However, some of the samples have been not permeable. Therefore we notice the skewed character of the porosity distribution (see Fig. 3, left). As a consequence, the tortuosity distribution is skewed as well, with most of the systems at $T<1.5$. We found a few systems with $T>2.0$ at low permeability, but they have been rejected from the final analysis because the predictions of the LBM solver are uncertain in this range. Moreover, to improve the learning process for higher porosity values, we generated additional samples with porosity $\varphi >0.85$. As a result, an important fraction of the samples belongs to the tail of the distributions. Training the network with such distributed data leads to the model with excellent performance on the data from the peak of distribution, and with the low predictive ability, for the systems from the tail of the distribution. We partially solve this problem by considering the re-weighted distribution of samples, namely,

**Table 1:** The ranges of the porosity, permeability, and tortuosity obtained for the ensemble of 100,000 samples of the systems generated within the fluid flow simulation procedure.

	Porosity	Permeability	Tortuosity
Min	0.37	0.78	1.03
Max	0.99	$2.1\times 10^5$	2.74

- the data are binned in the two-dimensional histogram in the permeability and tortuosity;
- for every bin we calculate the weight given by the ratio of the total number of samples to the number of samples in a given bin;
- the ratios form the re-weighted distribution of the samples;
- during the training, labeled figures in the mini-batch, are sampled from the re-weighted distribution.

Another standard procedure, adapted by us, is the transformation of labels, so that, they have the values on the neighborhood of zero.

6 $$\begin{aligned} \varphi\rightarrow & {} \varphi /\varphi _{s} - {\overline{\varphi }} \end{aligned}$$

7 $$\begin{aligned} T\rightarrow & {} T/T_{s} - {\overline{T}}. \end{aligned}$$

In the case of the permeability, instead of *k*, we consider $\log$ of *k* and do re-scaling:

8 $$\begin{aligned} k \rightarrow \log (k)/\log (k_s) - \log ({\overline{k}}). \end{aligned}$$

In the final analyses the following settings are used: $\varphi _{s}=1.0$, ${\overline{\varphi }}=0.5$, $T_{s}=2.8$, ${\overline{T}}=0.5$ as well as $\log (k_s)=12.3$, $\log ({\overline{k}}) = 0.5$.

**Table 2:** The mean of ${\overline{R}}$ and $\sqrt{Var(R)}$ computed from the predictions of networks $net_A$ and $net_B$.

	$\varphi$	*k*	*T*
$$\varvec{net}_{\varvec{A}}$$			
Training set			
${\overline{R}}$	− 5.27e−04	− 8.63e−04	− 1.65e−04
$\sqrt{Var(R)}$	9.40e−03	7.54e−02	2.47e−03
Validation set			
${\overline{R}}$	− 5.83e−04	− 8.88e−04	− 1.37e−04
$\sqrt{Var(R)}$	9.33e−03	5.55e−02	2.45e−03
$$\varvec{net}_{\varvec{B}}$$			
Training set			
${\overline{R}}$	− 2.64e−04	− 1.91e−04	2.81e−06
$\sqrt{Var(R)}$	7.44e−03	3.35e−02	2.01e−03
Validation set			
Mean	− 3.64e−04	− 1.48e−03	− 1.70e−05
$\sqrt{Var(R)}$	7.31e−03	6.28e−01	2.07e−03

*R* is defined by Eq. (11).

### Network architecture

In order to define the CNN architecture we introduce the blocks:

- *C*(*N*, *K*, *S*, *P*, *act*) convolutional layer with: *N* kernels of the size $K\times K$ with the stride *S*, padding *P* and *act* - activation function;
- $MP(K=2) \equiv MP()$ max pooling layer of the size $K\times K$;
- *B*()—batch normalization layer;
- *F*(*M*, *act*) fully connected layer with activation function *act*.

The code has been implemented using the PyTorch library^47^. We distinguish two types of the analyses:

(A) the input figures are re-sized to $200\times 200$;
(B) the input figures are original of size $400 \times 400$.

In the first case, the training of the network is faster, however, a fraction of the information, hidden in the figures, might be lost. In the other, the training is slower, but full information is utilized in the analysis.

**Figure 4:**
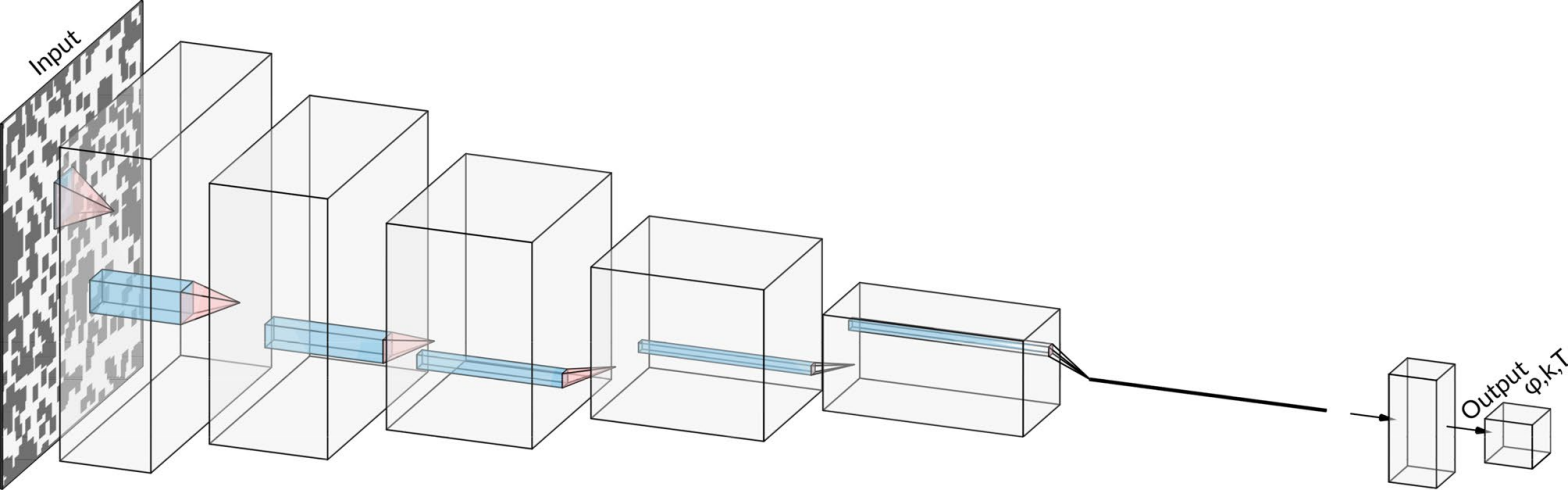
The network $net_{A}$ architecture. It contains six convolutional blocks and two (including output) fully connected layers. Each convolutional block contains the max pooling layer. The first three convolutional blocks consist of the batch normalization layers. The graph was drawn using NN-SVG^46^.

For the analysis (A) we consider a network $net_A$. It contains six convolutional blocks and two (including output) fully connected layers (see Fig. 4), namely:

9 $$\begin{aligned} input\rightarrow & {} C(10, 10, 1, 0, \mathrm {ReLU})\cdot B() \cdot MP() \nonumber \\\rightarrow & {} C(20, 7, 1, 0, \mathrm {ReLU})\cdot B() \cdot MP() \nonumber \\\rightarrow & {} C(40, 5, 1, 0, \mathrm {ReLU})\cdot B() \cdot MP() \nonumber \\\rightarrow & {} C(80, 3, 1, 0, \mathrm {ReLU})\cdot MP() \nonumber \\\rightarrow & {} C(160, 2, 1, 0, \mathrm {ReLU})\cdot MP() \nonumber \\\rightarrow & {} C(400, 2, 1, 0, \mathrm {ReLU})\cdot MP() \nonumber \\\rightarrow & {} F (10,\mathrm {\tanh }) \nonumber \\\rightarrow & {} F (3,id) = output, \end{aligned}$$

where *id* is identity map and $\tanh$ refers to hyperbolic tangent.

The network, $net_B$, used in the analysis (B), contains seven convolutional blocks and two (including output) fully connected layers, namely:

10 $$\begin{aligned} input\rightarrow & {} C(10, 5, 1, 2, \mathrm {ReLU}) \cdot MP() \nonumber \\\rightarrow & {} C(10, 10, 1, 0, \mathrm {ReLU})\cdot B() \cdot MP() \nonumber \\\rightarrow & {} C(20, 7, 1, 0, \mathrm {ReLU})\cdot B() \cdot MP() \nonumber \\\rightarrow & {} C(40, 5, 1, 0, \mathrm {ReLU})\cdot B() \cdot MP() \nonumber \\\rightarrow & {} C(80, 3, 1, 0, \mathrm {ReLU})\cdot MP() \nonumber \\\rightarrow & {} C(160, 2, 1, 0, \mathrm {ReLU})\cdot MP() \nonumber \\\rightarrow & {} C(400, 2, 1, 0, \mathrm {ReLU})\cdot MP() \nonumber \\\rightarrow & {} F (10,\mathrm {\tanh }) \nonumber \\\rightarrow & {} F (3,id) = output \end{aligned}$$

Notice that the first three CNN blocks in $net_A$ and three subsequent blocks (starting from second) in $net_B$ contain the batch normalization layers.

### Training

The generated data set has been split into the training ($85\%$ of the total number) and validation ($15 \%$ of the total number) data sets. After the cut in the tortuosity, we have 84,917 and 14,986 samples in training and validation data sets, respectively.

In the pre-analysis, we considered two types of loss functions, namely, the mean absolute error (MAE) and mean square error (MSE). The MAE is more sensitive to the outliers. However, our preliminary experiments showed that optimization of the MSE leads to the models with better performance on the validation set than the models optimized with the MAE. Therefore, the final analysis has been run with the MSE loss. During each epoch step, we calculate the error on the validation data set. The model with the smallest error value is saved.

The stochastic gradient descent (SGD) algorithm, in the mini-batch version, has been utilized for the training^48,49^. The mini-batch contains 250 (analysis (A)) and 65 (analysis (B)) samples, respectively. SGD is one of the simplest learning algorithms. But it is known that it regularizes naturally the networks. As a result, the obtained models work better on the validation data set^20,50,51^.

## Results and summary

One of the difficulties in the DL analyses is a proper choice of the hyperparameters, such as the size of the mini-batch, the optimization algorithm parameters, number of the CNN blocks, the size of the kernels, etc. We experimented with various configurations of the hyperparameters. Eventually, we established the final settings, namely, the SGD algorithm is run with the momentum 0.9 and the initial value of the learning rate 0.1. The latter parameter is reduced by $10\%$ every 50 epoch of the training. The sizes of the mini-batches as well as network architectures are reported in the previous section.

Both network architectures are constructed so that the number of filters in the CNN increases, while the size of the inputs of the subsequent layers reduces with the depth of the network. The kernels in the first layer of the $net_B$ are relatively large ($K=10$). In the subsequent layers, the kernels are smaller. The $n_B$ is the network $net_A$ with an additional input layer. In the case of the $net_A$, instead of the layer with $K=10$, the input is re-sized. In both network schemes, (A) and (B), the output of the last CNN layer is a vector of the length 400.

**Figure 5:**
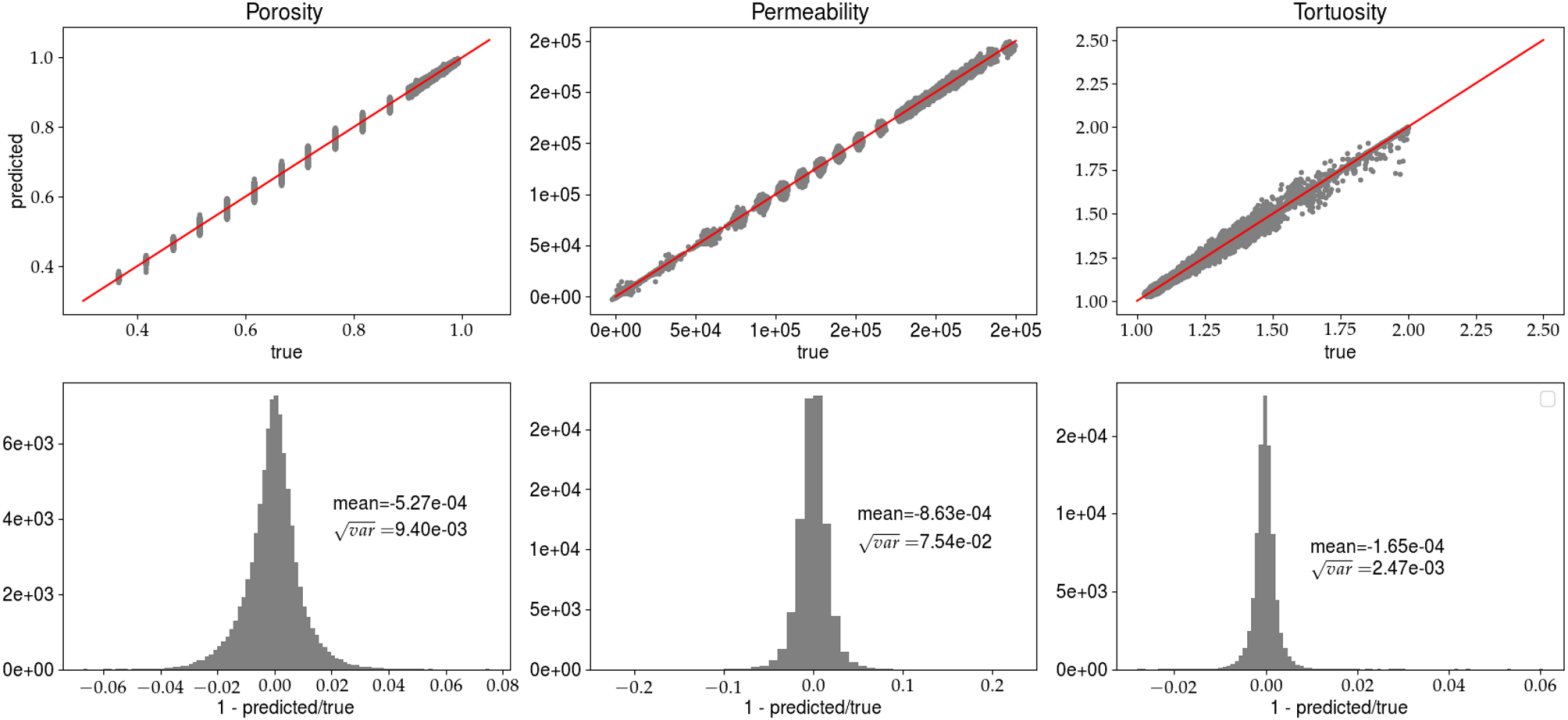
Predictions of the porosity, permeability, and tortuosity by the CNN versus ‘true‘ data (upper row). In the bottom row, the histograms of *R*, see Eq. (11), are plotted. The results are obtained for analysis (A) and the training data set. Solid line, in the top row, represents $predicted=true$ equality.

**Figure 6:**
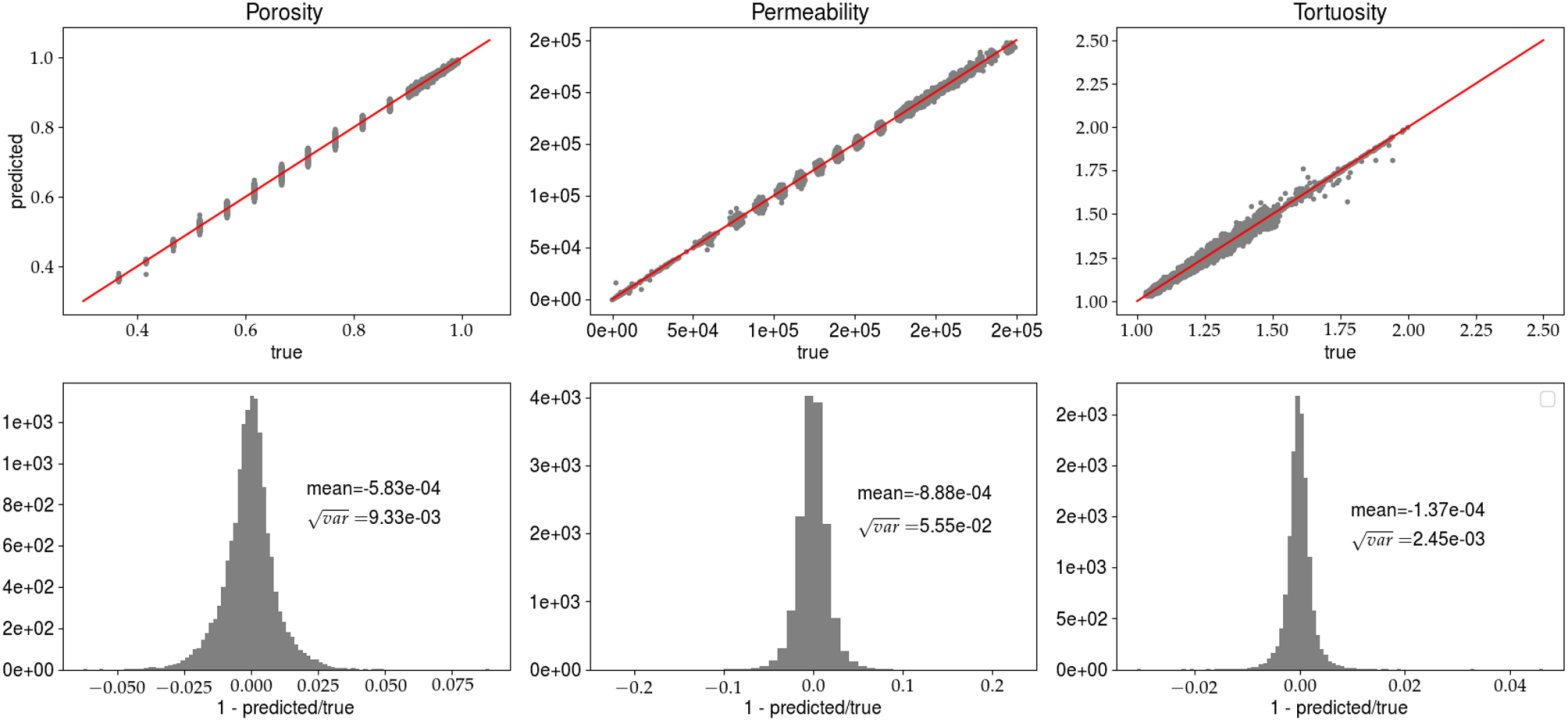
Caption the same as in Fig. 5 but the predictions are made for the validation data set.

**Figure 7:**
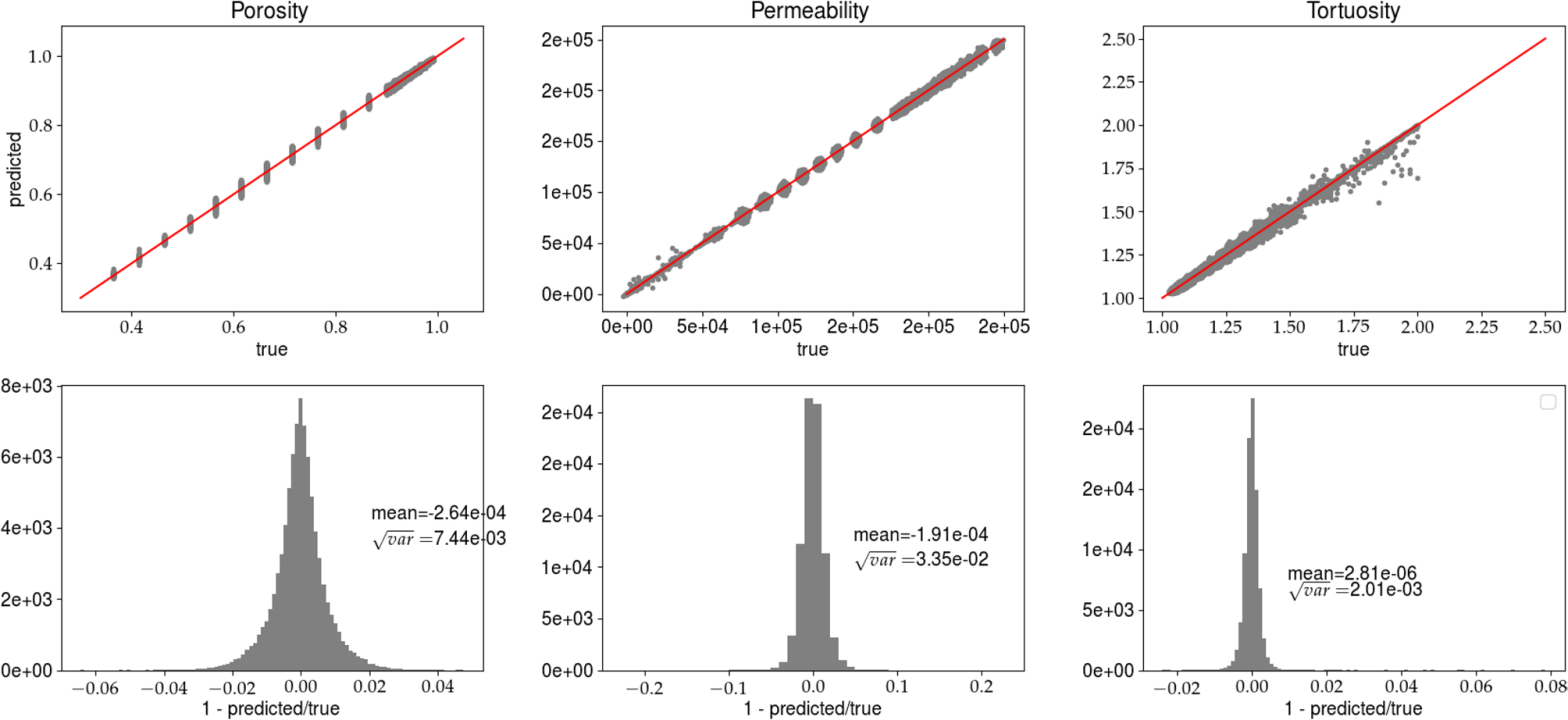
Predictions of porosity, permeability, and tortuosity by CNN versus ‘true‘ data (upper row). In the bottom row, the histograms of *R*, see Eq. (11), are plotted. The results are obtained for analysis (B) and the training data set. Solid line, in the top row, represents $predicted=true$ equality.

**Figure 8:**
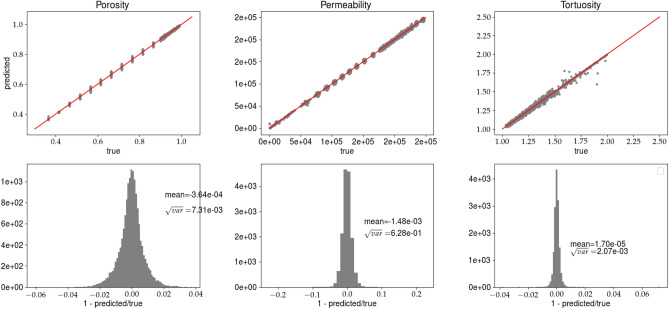
Caption the same as in Fig. 7 but the predictions are made for the validation data set.

The best model is the one with the smallest error on the validation set. Figs. 5 and 6 present the results for model $net_A$, whereas Figs. 7 and 8 show the results for the analysis (B). In each figure, the predictions of the porosity, permeability, and tortuosity versus the ‘true’ (as obtained from the LBM solver) values are plotted. Additionally, each figure contains the histograms of ratio

11 $$\begin{aligned} R = 1 - \frac{predicted}{true}, \end{aligned}$$

where *R* is computed for the porosity, permeability and tortuosity. More quantitative description of the histograms is given in Table 2, in which the mean (${\overline{R}}$) and variance $\sqrt{Var(R)}$ computed for all presented histograms are given. The variable *R* is more informative than the standard metrics MSE or MAE, which give only qualitative information about goodness of fit. Notice that we present the network predictions for the training and validation data sets.

The agreement between the network’s predictions and ‘true’ values seems to be pretty good. The performance of both networks is comparable. However, $net_A$, with the resized input, seems to work better on the validation data set. Both models predict porosity well. The difficulties appeared in modeling the permeability and tortuosity. Indeed, it was difficult to obtain accurate model predictions for the low permeability and the tortuosity $T>1.75$. The re-weighting procedure described above, allowed to partially solve this problem. Another improvement is given by including the batch normalization layers in the network architecture.

In the analysis (A), for a large fraction of samples the relative difference between the network response and the ’true’ value is smaller than $6\%$. The most accurate predictions are obtained for the porosity and tortuosity. Indeed, the difference between ’true’ and predicted is smaller than $1\%$. Model $net_B$ computes the porosity and tortuosity with similar accuracy as the model $net_A$, but the predictions of the permeability are rather uncertain in this case. The fact that the performance of both models is comparable shows that, in the case of porous media studied here, re-sizing of the input in the analysis (A) reduces only a small amount of information about the system. Moreover, model (A) has better performance on the validation data set than model (B). Hence, in our analysis, the re-sizing of the input figures works as a regularization of the model.

**Figure 9:**
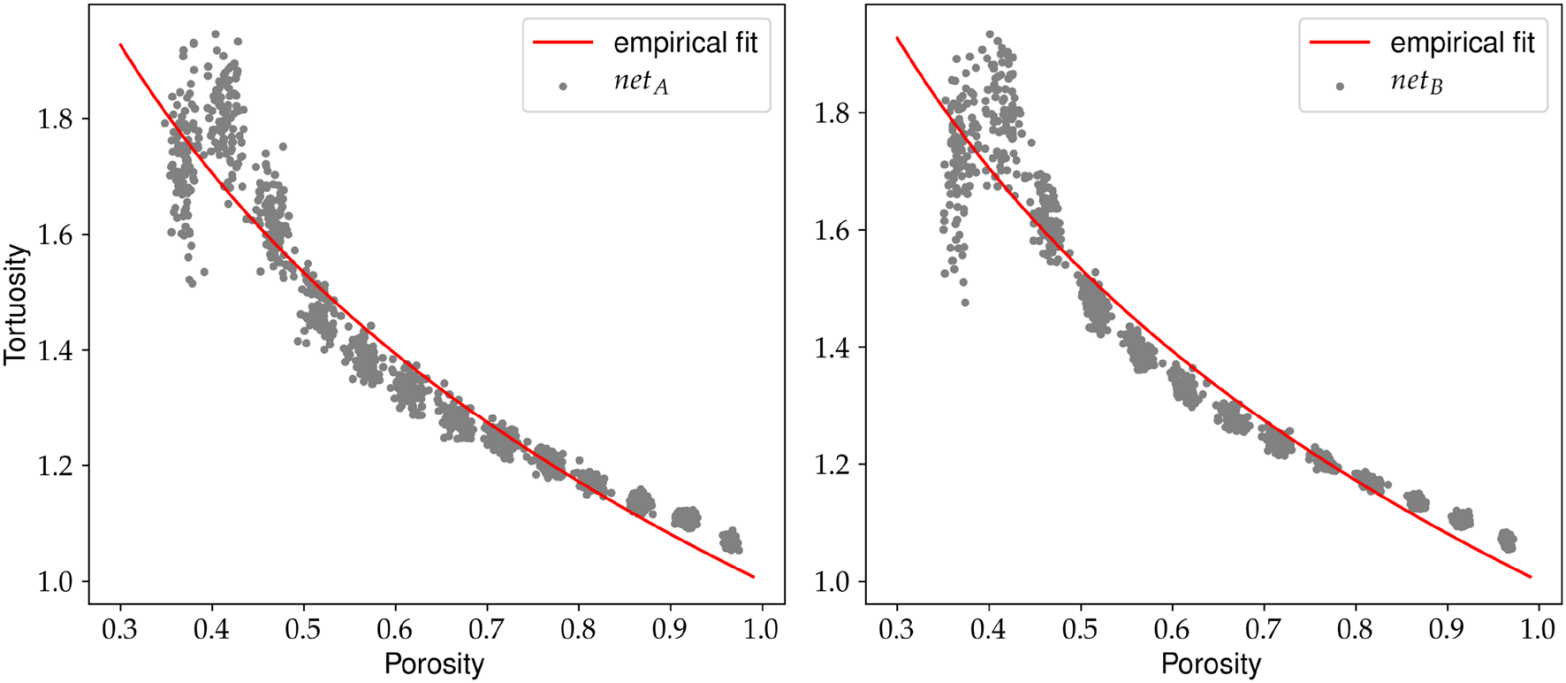
Tortuosity versus porosity: solid line represents empirical relation $T(\varphi )=1-0.77 \log (\varphi )$ obtained within classical fluid flow approach^12^. The points are obtained from the unlabelled data set.

Both data sets, training, and validation are used in the optimization process. Hence to examine the quality of obtained models we generated the third set of the data. It contains 1, 300 unlabelled samples. This data set is used to reconstruct the dependence between porosity and tortuosity, see Fig. 9). The obtained $T(\varphi )$ relation agrees with the empirical fit from the analysis of the fluid flow^12^ on qualitative level. Indeed, tortuosity grows with $\varphi \rightarrow \varphi _c$, where $\varphi _c$ is the percolation threshold ($\varphi \approx 0.4$ for overlapping quads model^52^). Due to the finite size of the considered system, the actual percolation threshold is not sharp, and tortuosity around $\varphi _c$ is underestimated in this area. The origin of the drop in the tortuosity below $\varphi _c$ is the finite size of samples.

To summarize, we have shown that the convolutional neural network technique is a good method for predicting the fundamental quantities of the porous media such as the porosity, permeability, and tortuosity. The predictions are made base on the analysis of the pictures representing the two-dimensional systems. The two types of networks have been discussed. In the first, the input pictures were resized, in the other, the input had the original size. We obtained good accuracy of predictions for all three quantities. The network models reproduce the empirical dependence between the tortuosity and porosity obtained in the previous studies^12^.
